# Growth of millimeter-sized 2D metal iodide crystals induced by ion-specific preference at water-air interfaces

**DOI:** 10.1038/s41467-024-47241-4

**Published:** 2024-04-12

**Authors:** Jingxian Zhong, Dawei Zhou, Qi Bai, Chao Liu, Xinlian Fan, Hehe Zhang, Congzhou Li, Ran Jiang, Peiyi Zhao, Jiaxiao Yuan, Xiaojiao Li, Guixiang Zhan, Hongyu Yang, Jing Liu, Xuefen Song, Junran Zhang, Xiao Huang, Chao Zhu, Chongqin Zhu, Lin Wang

**Affiliations:** 1 School of Flexible Electronics (Future Technologies) & Institute of Advanced Materials (IAM), Key Laboratory of Flexible Electronics (KLOFE), Jiangsu National Synergetic Innovation Center for Advanced Materials (SICAM), Nanjing Tech University (Nanjing Tech), Nanjing, 211816 China; 2 SEU-FEI Nano-Pico Center, Key Lab of MEMS of Ministry of Education, School of Integrated Circuits, Southeast University, Nanjing, 210096 China; 3 College of Chemistry, Key Laboratory of Theoretical & Computational Photochemistry of Ministry of Education, Beijing Normal University, Beijing, 100875 China

**Keywords:** Synthesis and processing, Two-dimensional materials, Synthesis and processing, Two-dimensional materials

## Abstract

Conventional liquid-phase methods lack precise control in synthesizing and processing materials with macroscopic sizes and atomic thicknesses. Water interfaces are ubiquitous and unique in catalyzing many chemical reactions. However, investigations on two-dimensional (2D) materials related to water interfaces remain limited. Here we report the growth of millimeter-sized 2D PbI_2_ single crystals at the water-air interface. The growth mechanism is based on an inherent ion-specific preference, i.e. iodine and lead ions tend to remain at the water-air interface and in bulk water, respectively. The spontaneous accumulation and in-plane arrangement within the 2D crystal of iodide ions at the water-air interface leads to the unique crystallization of PbI_2_ as well as other metal iodides. In particular, PbI_2_ crystals can be customized to specific thicknesses and further transformed into millimeter-sized mono- to few-layer perovskites. Additionally, we have developed water-based techniques, including water-soaking, spin-coating, water-etching, and water-flow-assisted transfer to recycle, thin, pattern, and position PbI_2_, and subsequently, perovskites. Our water-interface mediated synthesis and processing methods represents a significant advancement in achieving simple, cost-effective, and energy-efficient production of functional materials and their integrated devices.

## Abstract (web summary)

Here, the authors report the growth of millimetre-scale 2D PbI_2_ single crystals via the spontaneous accumulation and in-plane arrangement of iodide ions at the water-air interface. The metal iodide crystals could be thinned down to a single monolayer via water etching, and subsequently converted into perovskite optoelectronic devices.

## Introduction

The synthesis of large-scale two-dimensional (2D) materials, whether to enrich material variety or to develop method, is crucial for widespread applications. Water is widely recognized as critical for materials synthesis due to its abundance, cost-effectiveness, environmental compatibility, non-toxicity, and non-flammability. A growing body of research has demonstrated that many chemical reactions are significantly accelerated when occurring at water interfaces compared to the same reactions in the gas phase or bulk water^1–4^. This phenomenon is referred to as ‘on-water’ catalysis. However, there has been limited research on the controlled synthesis of two-dimensional (2D) materials, particularly on a large scale, at water interfaces. Two-dimensional (2D) materials are highly attractive for their potential in future integrated optoelectronic applications^5–7^. The pursuit of further advancements in this field drives the exploration of experimental strategies for synthesizing 2D semiconductors with macroscopic lateral dimensions, great optoelectronic properties, versatile material options, and strong sustainability. Despite great advances in vapor-phase growth methods for large-size 2D materials, the majority of research has predominantly focused on metallic graphene^8,9^, insulating boron nitride^10,11^, and limited types of semiconducting transition-metal dichalcogenides^12–14^. In contrast, liquid-phase growth, known for its ease of use, freedom from substrate constraints, and low energy consumption, has not yet been effectively employed for the production of large-size 2D crystals. In liquid environments, the non-directional motion of numerous ions leads to crystal growth characterized by excessive nucleation sites and random growth directions. Consequently, liquid-phase methods often result in 2D materials with small sizes, slow growth rates, and uncontrollable thicknesses. The liquid surface, however, offers a naturally confined 2D plane for ions to interact with each other laterally. Unfortunately, this has been mostly limited to the synthesis of organic or hybrid materials^15–17^. Additionally, such liquid-air interfacial growth typically relies on additives, which often introduces impurities or defects^18,19^. Considering the vital importance of water resources for human development and the fundamental role of water interfaces in scientific research, there is significant interest in identifying appropriate candidates for 2D materials and exploring growth mechanisms for water-air interfacial growth.

In this work, the synthesis of millimeter-sized 2D layered PbI_2_ single crystals at the water-air interface without any additive is performed and observed in situ. The success of this synthesis can be attributed to the ion-specific interfacial preference, where iodide ions (I^−^) naturally exhibit a strong affinity for the water surface. This growth mechanism stands in stark contrast to previously reported materials fabricated at the liquid surface through interfacial confinement effects. In addition, ultrathin halide perovskite materials that combine the high-integration potential for 2D materials and the great optoelectronic properties for halide perovskites have attracted dramatically increasing research attention^20–24^. A range of halide perovskites are available through the conversion of our-grown PbI_2_ crystals, of which the millimeter-scale lateral size is far ahead of previously reported mono- to few-layer halide perovskites.

## Results and discussion

### Water-air interfacial synthesis of PbI_2_

**Fig. 1:**
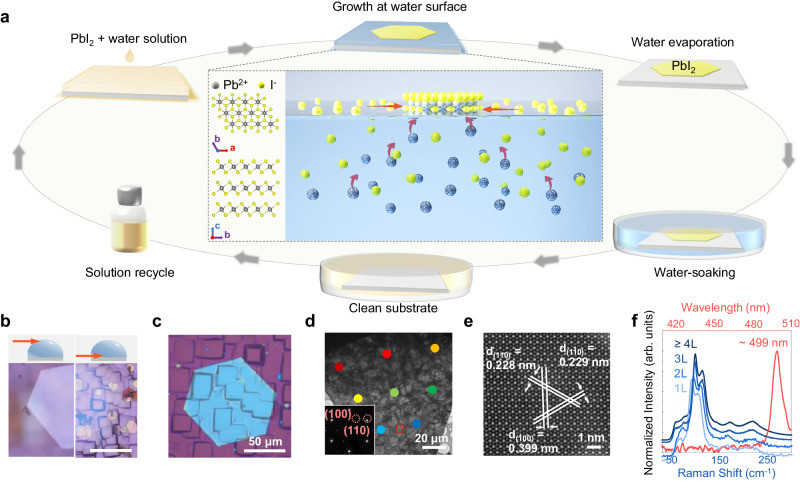
Large-size single-crystalline PbI_2_ grown at the water-air interface. **a** Schematic illustration of the crystal structure and the preparation, growth, and recycle process of 2D layered PbI_2_. The red arrows on the water surface indicate the horizontal accumulation of I^−^ and the red arrows in the water indicate the attraction of I^−^ to Pb^2+^. **b** Optical images of PbI_2_ when using the backside of Si/SiO_2_ as a substrate, with focal plane on water surface or substrate surface before and (**c**) after water evaporation. **d** Transmission electron microscopy and (**e**) scanning transmission electron microscopy images of a large-size PbI_2_ nanosheet obtained through water-air interfacial growth. The inset displays superimposed diffraction patterns captured at various regions of the nanosheet, denoted by different-colored dots, all of which closely coincide with each other, indicating the single crystalline structure of our-grown PbI_2_. **f** Micro-Raman (blue lines) and photoluminescence (red line) spectra of PbI_2_ at room temperature.

2D layered PbI_2_ with a bandgap of ~2.5 eV, has demonstrated fascinating potential in X-ray detection, memory, optoelectronics, and spintronics applications^25–34^. Figure 1a schematically depicts the water-based synthesis and recycle process of 2D PbI_2_ nanosheets. This simple synthesis, which only involves drop-casting PbI_2_ aqueous solution onto a substrate in ambient conditions, allows for the in-situ observation of the PbI_2_ growth process using optical microscopy (Supplementary Fig. 1). Notably, the entire nucleation and growth process of PbI_2_ nanosheets occurs at the water-air interface during water evaporation, with the nanosheets settling on the substrate surface once the water has completely evaporated. The nanosheets grow fast initially and stop growth before the complete evaporation of water, as similar to the typical crystallization from a supersaturated solution. While, the difference relies on that the crystallization at the water-air interface has a strong anisotropy, which facilitates the in-plane growth of 2D PbI_2_. Water-air interfacial growth is advantageous for freestanding materials due to minimal substrate requirements. To demonstrate this, we have successfully prepared the PbI_2_ crystals on the rough backside of SiO_2_/Si substrates, as illustrated in the Fig. 1b,c. Furthermore, we experimented with various substrates, including slides, parafilm, aluminum foil, CDs, leaves, and plastic wrap (Supplementary Fig. 2). Each substrate successfully produces PbI_2_ crystals, although not always with perfect quality (for reasons that will be discussed later). The substrates containing PbI_2_ crystals can be recycled by immersing them in water. Both the substrates and the waste water can then be used for the subsequent water-based growth of PbI_2_ nanosheets, respectively.

Most of these PbI_2_ nanosheets are hexagonal in shape, in consistence with the crystal structure of PbI_2_ (Fig. 1a). The crystal structure of the nanosheets is further experimentally identified by the transmission electron microscopy (TEM), Raman spectroscopy and X-ray diffraction characterizations (XRD). For TEM characterization, we can directly grow PbI_2_ nanosheets on TEM grids without any need for transfer or etching process, as benefited from the weak substrate-dependence for water-air interfacial growth method. As shown in Fig. 1d-e, the nanosheets exhibit uniform and high crystallinity, free from any observable grains or defects. This can be attributed to the single-point nucleation, which ensures that the large-size PbI_2_ nanosheet is single-crystalline; this is demonstrated by the overlapping diffraction patterns collected from various regions of the nanosheet. The TEM and XRD results (Supplementary Fig. 3), along with Raman and photoluminescence (PL) spectra (Fig. 1f), are consistent with 1T-phase PbI_2_ with the space group *P-3m2*^31,35^.

### Ion-specific interfacial preference

**Fig. 2:**
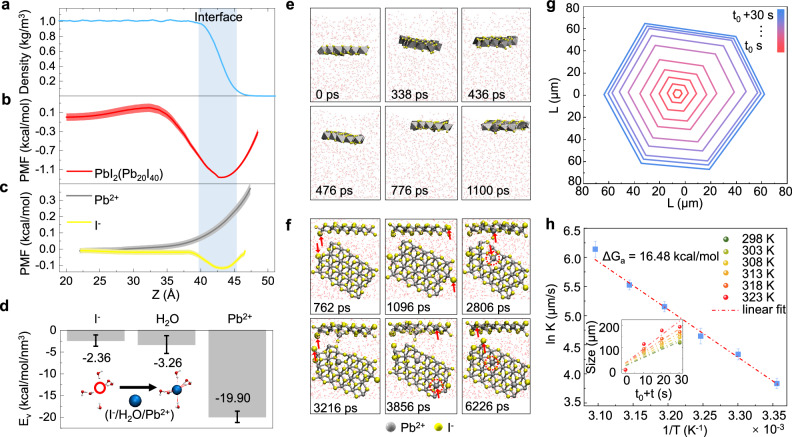
Water-air interfacial growth mechanism of PbI_2_. **a** Density of water molecules; The water-air interface is defined as the region between 90% and 10% of the bulk density (blue region). **b**, **c** Potential of mean force (PMF) profiles for the transfer of a monolayer PbI_2_ (**b**), Pb^2+^ ion and I^−^ ion (**c**) from the bulk water to the water-air interface. Standard deviation error bars are calculated from three independent trials with different initial structures. **d** Average interaction energy per unit volume (*E*_*v*_) of a water molecule or an ion with the surrounding water molecules in bulk water, where the error bars were calculated by calculating the root-mean-square based on the data of 100 configures. **e** Snapshot structures of the monolayer PbI_2_ nanosheet obtained from a classical molecular dynamics (MD) simulation, where the PbI_2_ nanosheet is initially placed in bulk water. **f** Time-lapse snapshots of MD simulations during the growth of the PbI_2_ nanosheet at the water-air interface. The small grey and yellow spheres represent the Pb and I atoms of a pre-existing PbI_2_ nanosheet, respectively. The large grey and yellow spheres represent the Pb^2+^ and I^−^ ions initially dissolved in water, respectively. Red arrows and dotted circles represent newly grown atoms. **g** Time-dependent contour plots of a PbI_2_ nanosheet grown at a temperature of 298 K (as extracted from Supplementary Movie 3). **h** Natural logarithm value of the growth coefficient (*K*) as a function of inverse temperature. The inset displays the size of PbI_2_ nanosheets as a function of time at temperatures *T* = 298 K, 303 K, 308 K, 313 K, 318 K, 323 K. The dot-dashed lines are linear fitting of the experimental data.

To investigate the growth mechanism of 2D PbI_2_ nanosheets at the water-air interface, we conducted calculations to determine the free energy profiles associated with the transfer of a monolayer PbI_2_, Pb^2+^ ion and I^−^ ion from the bulk water to the water-air interface (Fig. 2a-c). The free energy decreases by 1.3 kcal/mol as the monolayer PbI_2_ moves from the bulk water to the water-air interface, indicating a preference for the monolayer PbI_2_ to reside at the water-air interface. This preference for the PbI_2_ monolayer at the interface is further supported by classical molecular dynamics (MD) simulation (Fig. 2e and Supplementary Movie 1). When initially placed in the bulk water, the PbI_2_ nanosheet gradually migrates towards the water-air interface, in alignment with the experimental findings. Furthermore, free energy calculations reveal that I^−^ ions exhibit a strong affinity for the outer layer of the water-air interface region (Fig. 2b). In contrast, the free energy exhibits a monotonous increase as Pb^2+^ ions move from the bulk water to the water-air interface, suggesting that Pb^2+^ ions predominately remain within the bulk water (Fig. 2c). These distinct spatial distributions of I^−^ and Pb^2+^ ions in water promote the interfacial synthesis of ultrathin large-size PbI_2_ single crystals. Additionally, the interaction energy (*ΔE*_*b*_) between the introduced ions and the remaining PbI_2_ atoms reveals that I^−^ ions tend to attach to the in-plane edges of PbI_2_, while Pb^2+^ ions can attach to both the in-plane edge and the surface (Supplementary Fig. 4).

To gain insight into the mechanism driving the propensity of I^−^ ions towards the water-air interface, we performed calculations to determine the average interaction energy per unit volume (*E*_*v*_) between a water molecule or an ion and the surrounding water molecules in the bulk water. *E*_*v*_ is defined as

1 $${E}_{v}=\frac{{E}_{{all}}(X)-{E}_{{solvent}}(X)}{{V}_{{shell}}}$$

where ${E}_{{all}}(X)$ represents the total potential energy of the first hydration shell of *X* (*X* can be H_2_O, I^−^, or Pb^2+^), obtained from MD simulations. ${E}_{{solvent}}(X)$ is the potential energy of the same structure but without *X*, and ${V}_{{shell}}$ is the volume of the first hydration shell of *X*. As depicted in Fig. 2d, our calculations reveal that *E*_*v*_ for a water molecule is approximately −3.26 kcal/mol/nm^3^. In contrast, *E*_*v*_ for a Pb^2+^ ion is considerably lower, indicating a strong preference for Pb^2+^ to remain in the bulk. Conversely, *E*_*v*_ for an I^−^ ion is higher than that for a water molecule, signifying a preference for I^−^ to be located at the interface. Because of the enrichment of I^−^ ions at the water-air interface, they initially attach to the in-plane edges of PbI_2_, resulting in a negatively charged PbI_2_ structure. Subsequently, driven by electrostatic interactions, Pb^2+^ ions bind to the PbI_2_. This process leads to the in-plane growth of 2D PbI_2_. This proposed mechanism gains further validation through MD simulations of PbI_2_ growth at the water-air interface (Fig. 2f and Supplementary Movie 2). Even when Pb^2+^ ions attach to the surface of PbI_2_, they promptly reconfigure and form a perfect PbI_2_ crystal. In contrast, MD simulations reveal that PbI_2_ undergoes horizontal and vertical growth within the bulk water (Supplementary Fig. 5).

The growth mechanism, driven by the distinct spatial distributions of I^−^ and Pb^2+^ ions in water, finds further validation through control experiments involving the introduction of additional Pb^2+^ or I^−^ ions (Supplementary Fig. 6). Upon adding Pb(NO_3_)_2_ solute to increase the Pb^2+^ concentration from 2 mmol L^−1^ to 3 mmol L^−1^, the growth of PbI_2_ proceeds slowly, and no preference is observed at the water-air interface. However, when the I^−^ concentration is elevated from 4 mmol L^−1^ to 6 mmol L^−1^ by introducing KI solute, PbI_2_ growth accelerates significantly at the water-air interface, yielding large crystals. The presence of extra ions, whether Pb^2+^ or I^−^, leads to a notable reduction in PbI_2_ solubility. Nonetheless, our experiments demonstrate that additional Pb^2+^ ions have minimal impact on PbI_2_ growth. In contrast, a high concentration of I^−^ ions in the solution results in a more self-driven accumulation of I^−^ ions at the water-air interface, facilitating the in-plane growth of PbI_2_. Furthermore, we have successfully synthesized other metal iodides at the water-air interface, including SnI_2_, GeI_2_, and CdI_2_ (Supplementary Fig. 7). This further underscores the growth mechanism driven by iodide-ion interfacial preference and highlights the universality of our approach.

We further conducted real-time monitoring of the structural transformation of individual 2D PbI_2_ crystals within their natural growth environment (Supplementary Movie 3). The time-dependent contour map is illustrated in Fig. 2g, revealing that the growth profiles along different directions exhibit remarkable similarity, resulting in the consistent maintenance of a regular shape^36^. The inset in Fig. 2h presents the size evolution of PbI_2_ crystals as a function of time, with temperatures *T* = 298 K, 303 K, 308 K, 313 K, 318 K, and 323 K. Notably, the growth rate remains nearly constant at a fixed temperature but exhibits strong dependence on temperature itself. At room temperature (*T* = 298 K), the growth rate is measured at ~3.8 μm/s, whereas at 323 K, it increases to ~ 6.1 μm/s. This significant temperature dependence is reminiscent of an activated process. The growth rate *R* of the crystal is determined by the expression^37^

2 $$R=K(S-1)$$

where *K* represents the growth coefficient, and *S* = *m*/*m*_*s*_, with *m* as the concentration and *m*_*s*_ as the saturation concentration. Figure 2h displays a plot of ln *K* against the inverse temperature (1/*T*). The slope of this plot yields an activation energy of ∆*G*_*a*_ = 16.48 kcal/mol.

### Controllable synthesis of mono- to few-layer PbI_2_

**Fig. 3:**
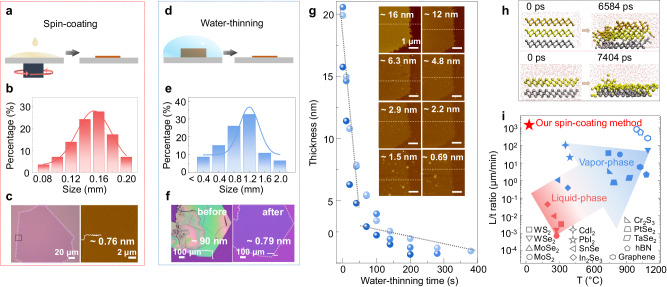
Controllable synthesis of mono- to few-layer PbI_2_. **a** Schematic demonstration, (**b**) statistical size distribution chart, and (**c**) optical and atomic force microscope (AFM) images of PbI_2_ nanosheets obtained through spin-coating. The solid lines are normal distribution fitting of the experimental data. **d** Schematic demonstration, (**e**) statistical size distribution chart, and (**f**) optical images of PbI_2_ nanosheets obtained through water-thinning. **g** Evolution of nanosheet thickness with water-thinning time. Inset show corresponding AFM images. The dotted lines are linear fitting of the experimental data. **h** Snapshots of the system obtained from classical molecular dynamics simulations of a trilayer (top panel) and a bilayer (bottom panel) PbI_2_ nanosheet on substrates in water. **i** Comparison of the growth temperature and the growth rate (lateral size L divided by time *t*) of monolayer 2D single crystals prepared using different methods based on single-point nucleation (solid symbols) and multi-point nucleation (open symbols)^10,12,38–53^.

With a deep understanding of the water-interfacial growth mechanism, we have further optimized the growth conditions and techniques to achieve precise control over the thickness of PbI_2_ down to monolayer. It is recognized that a droplet with reduced curvature offers an improved water-air interface conducive to the in-plane growth of PbI_2_. In light of this, we adopted a spin-coating approach after initially dropcasting a PbI_2_ aqueous solution droplet. Surprisingly, as illustrated in Fig. 3a-c, this spin-coating method not only significantly enhances the possibility of obtaining uniform PbI_2_ nanosheets with much thinner thicknesses, but also accelerates the growth rate. This acceleration is attributed to the increased evaporation rate for flat and thin droplets. The curvature of the droplet emerges as a crucial factor influencing PbI_2_ growth, further substantiated by the nanosheet morphology and contact-angle measurement results of the same PbI_2_ aqueous solution on SiO_2_/Si substrates but coated by different metals (Supplementary Fig. 8). These findings align with MD simulations of PbI_2_ nanosheets on the surface of water nanodroplets with varying contact angles. Notably, the first peaks of the radial distribution functions (RDFs) for I‐H_w_ and Pb‐O_w_ (H_w_/O_w_ representing H/O atoms in water) for the PbI_2_ nanosheet on the surface of water nanodroplets with a contact angle of 110° are higher compared to those with a contact angle of 70° (Supplementary Fig. 8). This observation indicates that the former exhibits a weaker surface preference compared to the latter. This also elucidates why various substrates can yield PbI_2_ crystals with distinct morphologies and thicknesses (Supplementary Fig. 2).

While the spin-coating method can produce thinner PbI_2_ nanosheets compared to the drop-casting method, it generally results in smaller lateral sizes. This is primarily due to the rapid evaporation of the solution and insufficient growth time during the spin-coating process. To combine the features of “millimeter-size” and “mono- to few-layer” into a single nanosheet, we have developed a water-thinning method that leverages the controllable water solubility of PbI_2_. In this method, substrates containing large but thick or visually uneven PbI_2_ nanosheets, prepared using the drop-casting method, are immersed in water. Alternatively, ethanol can be added to the water to slow down the thinning rate of PbI_2_ nanosheets. After a precisely controlled immersion period, the substrates are removed and quickly dried using a gas gun. As a result, the nanosheets can be thinned to the desired thickness determined by factors such as the immersion time, ethanol concentration, and initial thickness. For the nanosheets thicker than 90 nm, we can apply plasma pre-treatment before water-thinning to expedite the process. For instance, as shown in Fig. 3d–f, a very thick and nonuniform PbI_2_ nanosheet can be transformed into a uniform mono-layer through the water-thinning method. Moreover, the statistically measured lateral sizes for mono- to few-layer PbI_2_ nanosheets, prepared using the combined drop-casting and water-thinning method, are nearly one order of magnitude larger than those obtained by spin-coating method, as compared in Figs. 3a–c and 3d–f. Figure 3g displays the thickness evolution to tri- bi-, mono-layers of different nanosheets for varying water-thinning time. In this case, water containing a 50% concentration of ethanol is used to illustrate a gradual thinning-down process. The layer number of PbI_2_ can be identified through optical microscopy, AFM and Raman characterizations, as demonstrated in Fig. 1f^35^ and Fig. 3g, similar to other 2D materials. These characterization results consistently indicate that water-thinned PbI_2_ nanosheets maintain a complete shape and uniform surface regardless of variation in water-thinning time. In general, achieving precise control over a liquid-involved process to achieve high-quality flat morphology down to a monolayer is highly challenging. The success of the water-thinning method for PbI_2_ nanosheets relies on their layer-by-layer solubility in water. In order to gain a deeper understanding of the underlying mechanism, we conducted molecular dynamics (MD) simulations to investigate the dissolution behavior of bi-layer and tri-layer PbI_2_ nanosheets on a substrate in water. As depicted in Fig. 3h, the bottom layer of PbI_2_ adjacent to the substrate remains highly stable for both bi-layer and tri-layer PbI_2_ nanosheets, whereas the uppermost layer of PbI_2_ has already partially dissolved at t = 7404 ps and *t* = 6584 ps for the bi-layer and tri-layer PbI_2_ nanosheets, respectively. These simulation results suggest that PbI_2_ nanosheets on a substrate in water dissolve layer by layer from top to bottom, and the PbI_2_ bottom layer, characterized by strong interfacial interaction with the substrate, possesses a unique water-resistant property. This property facilitates the controlled thinning of a thick crystal down to a monolayer.

Typically, higher temperatures tend to result in faster crystal growth. In this context, the growth of PbI_2_ at the water-air interface stands out with a growth rate of up to 1300 µm/min, achieved at the lowest growth temperature down to 25 °C (Fig. 3i), which surpasses the findings in the literatures regarding the synthesis of monolayer 2D single crystals. Additionally, our monolayer PbI_2_ crystals reach an impressive size up to 2000 µm, a remarkable achievement when compared to previously reported synthesis of PbI_2_ single crystals (Supplementary Fig. 9). It is important to highlight that our method is unique due to its ease of operation, energy efficiency, and minimal demands on substrates or equipment. Vapor-phase methods often necessitate high-temperature process (>250 °C) and specific types of substrates, while liquid-phase methods typically yield small-size crystals (<1 µm) with long growth time^10,12,38–53^. We achieve the solution-processed synthesis of 2D layered inorganic crystals exceeding than 100 μm in size, especially without the use of any additional chemicals^15–19,39–41,54^.

### Conversion to mono- to few-layer perovskites

**Fig. 4:**
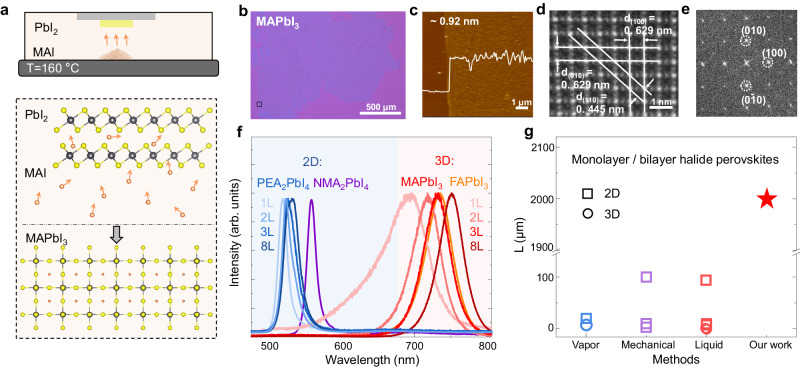
Synthesis of millimeter-sized mono- to few-layer perovskites. **a** Schematic illustration of the PbI_2_ to perovskite conversion. **b** Optical image, (**c**) atomic force microscope image, (**d**) transmission electron microscopy image, and (**e**) selected area electron diffraction of the millimeter-sized monolayer MAPbI_3_ nanosheets. **f** Photoluminescence spectra of 2D perovskites PEA_2_PbI_4_ and NMA_2_PbI_4_, as well as 3D perovskites MAPbI_3_ and FAPbI_3_. The blue area represents 2D perovskite and the red area represents 3D perovskite. **g** Comparison of the lateral size of monolayer and bilayer halide perovskites prepared using our method and other methods^17,55–63^. The terms of “vapor”, “mechanical”, and “liquid” stand for vapor-phase, mechanical exfoliation, and liquid-phase methods, respectively.

PbI_2_ is a most popular growth precursor for halide perovskite, an emerging semiconductor family with exotic properties^20,21^. Utilizing PbI_2_ nanosheets as growth templates, we can further efficiently produce a series of large-size mono- to few-layer lead iodine perovskites. The conversion method of mono- to few-layer PbI_2_ into perovskite is no need of furnace equipment anymore and can be conducted on a hotplate in ambient conditions. This can be attributed to the ultrathin nature of these PbI_2_ templates, enabling the conversion to perovskites under mild conditions. As depicted in Fig. 4a, organoiodized salt powders are placed on a hotplate, while the PbI_2_ nanosheets are positioned at the bottom of an inverted glass petri dish. The organoiodized salts are vaporized at relatively higher temperature and subsequently enter the PbI_2_ nanosheets, facilitating the perovskite conversion process. Figure 4b, c showcases the morphology of a typical monolayer MAPbI_3_ perovskite nanosheet with a lateral size of ~ 2 mm and a thickness of ~ 0.92 nm. The successful PbI_2_-perovskite conversion is further validated through TEM (Fig. 4d), SAED (Fig. 4e), PL (Fig. 4f), and XRD (Supplementary Fig. 3). In comparison with previous research efforts^17,55–63^, our method yields the largest lateral sizes for mono- to few-layer perovskites (Fig. 4g). Moreover, our method can not only be applied to layered 2D perovskites like PEA_2_PbI_4_ and NMA_2_PbI_4_ with long-chain organic cations, but also to non-layered 3D ones such as MAPbI_3_ and FAPbI_3_ with short-chain organic cations. Regardless of the perovskite composition, our method consistently produces perovskites with strong photoluminescence (PL), with intensities orders of magnitude higher than those observed in other 2D semiconductors of similar thickness, which suggests potential for future applications in low-power nanoscale optoelectronics^22,64^.

### Water-based device processing

**Fig. 5:**
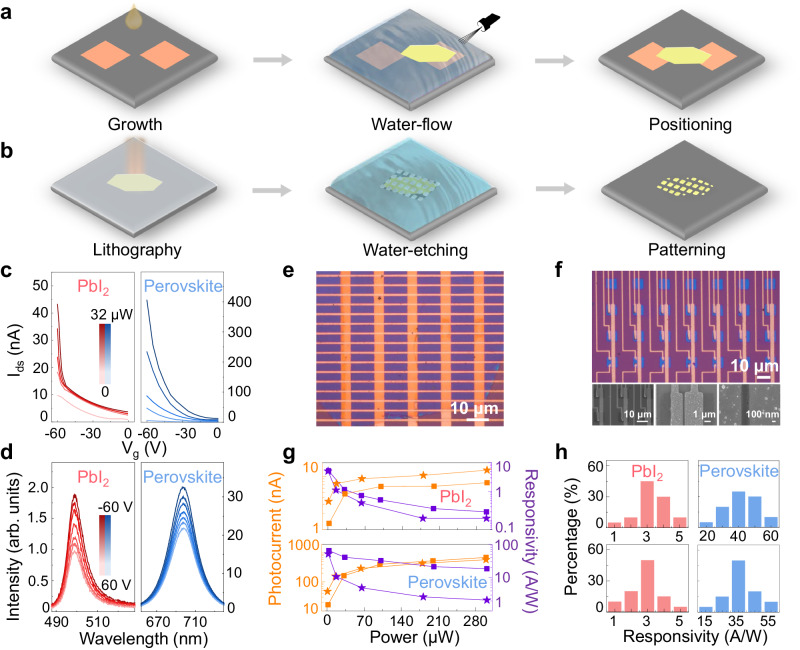
Device fabrication and performance of PbI_2_ and related perovskites through water-assisted techniques. **a** Schematic flow of the water-flow-assisted transfer technique for positioning PbI_2_. **b** Schematic flow of the water-etching technique for patterning PbI_2_. **c** Transfer curves of PbI_2_ and MAPbI_3_ devices under 405-nm light illumination with varying laser powers at bias voltage (*V*_*ds*_*)* = 2 V. **d** Gate-tunable photoluminescence spectra of PbI_2_ and MAPbI_3_ under 405-nm light illumination. **e** Optical image of a large-size PbI_2_ device with cross-array architecture. **f** Optical image and zoom-in scanning electron microscope images of highly integrated planar devices based on patterned PbI_2_. **g** Champion photocurrent and photo-responsivity results of PbI_2_ and MAPbI_3_ devices under 405-nm light illumination with cross-array architecture (star symbol) and planar devices (square) at *V*_*ds*_ = 2 V. **h** Statistical distribution chart of PbI_2_ and MAPbI_3_ photo-responsivities under 405-nm light illumination with cross-array architecture (upper panel) and planar devices (bottom panel) at *V*_*ds*_ = 2 V.

Water is a rich and clean resource with multi-degree of freedom, which can not only facilitate us in material growth, but also in device fabrication. The fabrication process relies on the precise control of material positioning and patterning to achieve desired properties, traditionally, of which the methods are always isolated from that of growth. Inspired by the water-air interfacial growth and the water fluidity, we have developed a method to seamlessly integrate material growth and positioning for PbI_2_. To elaborate, as illustrated in Fig. 5a, we dropcast a PbI_2_ aqueous solution onto the target substrate, resulting in the formation of PbI_2_ nanosheets. The position of a selected PbI_2_ nanosheet can be adjusted by controlling the direction of water-flow using gas-flow. Once the water has dried, the selected PbI_2_ nanosheet can fall and make contact with pre-designed structures or materials. This method ensures a clean and uniform interfacial contact, as it does not require the use of any additives or polymers throughout the integrated process of growth and transfer. While this water-based transfer method has lower position precision (~ 10 µm) compared to conventional dry-transfer methods, where the position of solids can be precisely controlled, it completely eliminates the common issues such as cracks, wrinkles, or contaminants that frequently arise during conventional dry-transfer processes^65^.

In addition to positioning, patterning is another important technique in device fabrication. Typically, material patterning is achieved through either liquid or dry etching processes, employing chemical liquids or reactive gases as etchants, respectively. Differently, we have devised a clean, cost-effective, and simple approach to patterning PbI_2_, utilizing water as the sole etchant. Following a standard lithography process, we pattern the spin-coated resist layer covering PbI_2_, which then serves as a mask in the water-etching process (Fig. 5b). The unprotected areas of PbI_2_ can be efficiently removed by immersion in a large volume of water for an extended period. As a result, desired structural patterns are imparted onto PbI_2_, all while maintaining a pristine surface without the need for corrosive chemicals or high-energy processes.

These two water-based techniques offer significant advantages in the fabrication of PbI_2_ and related perovskite devices. Notably, they address the compatibility issues that often arise between perovskites and conventional nanofabrication methods, particularly due to the vulnerability of halide or hybrid perovskites to damage when exposed to organic solutions. First, owing to their ultrathin thickness, both PbI_2_ and perovskite (demonstrated here with MAPbI_3_) devices exhibit a strong gate modulation effect in electronic transport and PL measurements. The transfer curves shown in Fig. 5c reveal that both PbI_2_ and MAPbI_3_ exhibit p-type semiconductor characteristics. While, when subject to light illumination, MAPbI_3_ demonstrates significantly enhanced conductivity compared to PbI_2_, primarily due to the light absorbing properties of perovskites. Regarding gate-tuned PL, the intensity of both PbI_2_ and MAPbI_3_ increases as the gate voltage is reduced, as depicted in Fig. 5d. This observation implies that, when reduced to ultrathin thicknesses, PbI_2_ and perovskites materials can be endowed with additional means of control, akin to other 2D materials. This provides them with significant potential for applications involving multiple physical fields and interfacial engineering^66,67^.

Furthermore, owing to their large size, the water-based fabrication techniques enable the creation of integrated circuited systems with various configurations. Figure 5e showcases a cross-array structured device, while Fig. 5f illustrates the potential for designing planar devices utilizing flexibly patterned PbI_2_ and related perovskites. All of these devices demonstrate excellent photodetection performance, surpassing previous achievements (as depicted in Fig. 5g,h)^27,29,68,69^. This clearly highlights the synergistic benefits of water-air interfacial growth and water-based fabrication techniques, offering us with good material quality and superior device performance.

The synthesis and processing of large-size 2D materials is crucial for industrial production and widespread applications, whether to enrich material variety or to develop method. In this study, we present the demonstration of 2D inorganic crystals at the liquid surface, especially utilizing the most cost-effective, abundant, and readily available water source, without any additives. This achievement is based on a growth mechanism referred to “ion-specific preference at water interfaces”, where iodine ions naturally accumulate at the water-air interface and react with crystals in the in-plane direction. Consequently, these crystals can grow up to millimeter-scale lateral sizes and down to monolayer thickness, with the fastest growth rate reaching up to 1300 μm/min at a lowest growth temperature of 25 °C. Furthermore, this method enables the production of mono- to few-layer halide perovskites with large lateral size by further intercalation of organic molecules. The utilization of water-based processing techniques, including water-soaking, spin-coating, water-etching, and water-flow-assisted transfer methods, facilitates the fabrication of metal iodide materials and devices with environmentally friendliness, high-integration, and high-performance.

## Methods

### Growth of PbI_2_ and related perovskites

First, the PbI_2_ aqueous solution with a concentration of 0.002 mol/L was drop-casted onto the substrate in an open ambient environment at room temperature. Multiple PbI_2_ nanosheets appeared and floated on the surface of the droplet within a time of seconds, eventually setting on the substrate once solution had dried. For the spin-coating method, the substrate was spin-coated with a speed of 2000 rpm for 10 s after drop-casting PbI_2_ aqueous solution. For the water-thinning method, a plasma pre-treatment for around 60 s is required to expedite the crystal thinning process when dealing with thick or ununiform initial crystals. For thinner crystals, the addition of ethanol to the water proves helpful for precise control of crystal thickness. PbI_2_ nanosheets can be further transformed to a range of perovskites by a simple heating table method, such as PEA_2_PbI_4_, NMA_2_PbI_4_, MAPbI_3_ and FAPbI_3_. The evaporation temperature for PEA_2_PbI_4_, NMA_2_PbI_4_, MAPbI_3_ and FAPbI_3_ is 190 °C, 150 °C, 160 °C and 180 °C, respectively.

### Characterizations

Optical microscope images were captured using a Nikon ECLIPSE Ci microscope. Scanning Electron Microscope (SEM) images were acquired by a JSM-7610F PLUS operating at 1 kV. Transmission Electron Microscope (TEM) images were obtained by a JEOL ARM200F at 200 kV. Crystal structures were analyzed through powder XRD analysis conducted on a Smart lab Rigaku instrument. Height profiles were measured using AFM (Asylum Research MFP-3D Origin) in noncontact mode. Raman spectra were recorded under 532-nm excitation with an output of 10 µW, while PL spectra were recorded under 405 nm excitation with 1 µW, where the laser beam was focused to a spot size of ≈2 µm (Zolix Finder Smart FST2-MPL501-405C1). All photoelectric measurements were performed using a Keithley 2612B instrument. Unless specified otherwise, all the measurements were performed at room temperature.

### Water-flow assisted positioning of PbI_2_

The electrodes (Au = 30 nm) were fabricated using a standard lithography process. Subsequently, the PbI_2_ aqueous solution was drop-casted onto the substrate with the electrodes to g multiple PbI_2_ nanosheets. Once the target PbI_2_ nanosheet of interest was identified, its position on the water surface was adjusted by controlling the gas flow direction, achieved using either a syringe or a gas gun. Following the drying of the solution, the target PbI_2_ nanosheet can be directed to drop onto the designed electrodes.

### Water-etching assisted patterning of PbI_2_

Standard electron-beam lithography (EBL) process was performed on polymethyl methacrylate (PMMA) spin-coated PbI_2_ nanosheets, followed by soaking in developer solution (isopropyl alcohol) for 1 min and in ultra-pure water for 2 min. The PMMA resist protected certain regions of the PbI_2_, while the unprotected areas were entirely etched away by water. As a result, the resist patterns were effectively transferred onto PbI_2_.

### Fabrication of PbI_2_ and perovskite devices

For the planar configuration, standard EBL process was performed on the patterned PbI_2_, followed by the thermal evaporation of 30-nm-thick Au as electrodes. After performing photoelectric measurements on the PbI_2_, the channel can be converted into corresponding perovskites for further perovskite measurements. For the vertical configuration, bottom electrodes were first fabricated using a standard lithography process. Then, a large-size PbI_2_ nanosheet was transferred by water-flow assisted positioning technique. Subsequently, the top electrodes were fabricated once again using the standard lithography process.

### Molecular-dynamics (MD) setup

Most of our liquid water models consisted of 5611 water molecules placed within a simulation box of 49.7856 × 49.7856 × 100 (x × y × z) Å^3^. Water was modelled using the transferable intermolecular potential 3 point (TIP3P) model, which is defined by the sum of a long-range Coulomb potential and a short-range Lennard-Jones (LJ) potential. The MYP1 interatomic model was used for PbI_2_, with parameters for water-PbI_2_ interaction chosen as referenced in ref. ^70^. We employed the Particle-particle particle-mesh (PPPM) algorithm to calculate the long-range interactions, with a cutoff of 1.0 nm applied for the Lennard-Jones potential. Periodic boundary conditions were applied in all three directions, and the water-air interface was oriented perpendicular to the z-axis.

MD simulations were conducted in a constant-volume and constant-temperature (*NVT*) ensemble, where the temperature (298 K) was regulated by the Nose-Hoover thermostat. The Verlet algorithm was employed to numerically integrate Newton’s equations of motion, with a time step of 1 fs. All MD simulations were executed using the Large-scale Atomic/Molecular Massively Parallel Simulator (LAMMPS) package.

### Free-energy calculations

The free energy profiles for the transfer of a PbI_2_ nanosheet (Pb_20_I_40_), a Pb^2+^ ion, or an I^−^ ion from the water-air interface into bulk water were computed using the umbrella sampling method integrated with MD simulations. To calculate the free energy of I^−^ and Pb^2+^ ions, one Na^+^ and two NO_3_^-^ were introduced to the system as compensating ions, respectively. In each sample window, a harmonic potential of 0.2 eV/nm^2^ was applied along the z-component between the center of mass (COM) of the PbI_2_ nanosheet, Pb^2+^ or I^−^ ions, and the COM of the water slab. The system was equilibrated for 1 ns in each window, and the final 0.9 ns of data were utilized to derive the free energy profiles through the weighted histogram analysis method (WHAM)^71^.

### MD simulations of mono-layer PbI_2_ nanosheets growth from solution

Two drastically different initial positions for the monolayer PbI_2_ nanosheets were considered. Specifically, the monolayer PbI_2_ nanosheets were initially positioned either at the water-air interface or within the bulk water. For the monolayer nanosheets initially placed within the bulk water, the z-coordinates of all atoms within the nanosheet were fixed. The Pb^2+^ ions were initially situated in the bulk water, while the I^−^ ions were located at the water-air interface. Every 3 ns, we introduced one Pb^2+^ and two I^−^ ions to the simulation cell.

### Study of water-air preference of PbI_2_ nanosheets

To delve deeper into the water-air preference of PbI_2_ nanosheets, we conducted MD simulations to study the solvation of PbI_2_ nanosheets in water. Initially, the PbI_2_ nanosheets were situated within the bulk water. The total simulation time was 5 ns.

### Insight into the stability of PbI_2_ nanosheets on substrate in water

MD simulations were performed to investigate the stability of PbI_2_ nanosheets on a substrate immersed in water. As illustrated in Fig. 3h, a bilayer or trilayer PbI_2_ nanosheet was placed on a smooth surface within the water environment. The water-smooth surface and the PbI_2_-smooth-surface interactions were modeled using the 9-3 LJ potential in the form $E=4\varepsilon [{\left(\sigma /r\right)}^{9}-{\left(\sigma /r\right)}^{3}]$, where *r* is the distance from the wall to the atoms. The interaction parameters used were ${\varepsilon }_{{{{{{{\rm{P}}}}}}}_{{{{{{\rm{b}}}}}}}{{{{{\rm{S}}}}}}}=0.16$ eV/Å^2^, ${\sigma }_{{{{{{{\rm{P}}}}}}}_{{{{{{\rm{b}}}}}}}{{{{{\rm{S}}}}}}}=3.2$Å, ${\varepsilon }_{{{{{{\rm{IS}}}}}}}=0.16$ eV/Å^2^, ${\sigma }_{{{{{{\rm{IS}}}}}}}=$ 3.2 Å. To accelerate MD simulations, the temperature *T* was maintained at 360 K.

### Interaction of PbI_2_ nanosheets with the surface of water nanodroplets

MD simulations were performed to investigate the interaction of PbI_2_ nanosheets with the surface of water nanodroplets with different contact angles. These nanodroplets consisting of 3828 water molecules were placed on a smooth surface. The water-smooth surface interaction was modeled by the 9-3 LJ potential, with surface 1 having parameters ${\sigma }_{{{{{{\rm{WS}}}}}}}=$ 3.2 Å and ${\varepsilon }_{{{{{{\rm{WS}}}}}}}=$0.02 eV/Å^2^, and surface 2 with ${\sigma }_{{{{{{\rm{WS}}}}}}}=$ 3.2 Å and ${\varepsilon }_{{{{{{\rm{WS}}}}}}}=$0.01 eV/Å^2^. The contact angles were determined to be 70 ° and 110°, respectively. Both systems underwent an initial pre-equilibration phase for the first 3 ns at 300 K in an *NVT* ensemble. Subsequently, monolayer PbI_2_ nanosheets were introduced at the water-air interface of the water nanodroplets, and production trajectories were obtained from 3 ns simulations conducted in an *NVT* ensemble.

### Formation energies of ions on the surface or in-plane edges of PbI_2_ nanosheets

We have calculated the formation energies for varying numbers of I^−^ (or Pb^2+^) ions positioned on either the surface or the in-plane edges of PbI_2_ nanosheets. The formation energy is defined as follows:

3 $$\triangle E({{{{{\rm{N}}}}}})=E\left[{\left({ion}\right)}_{N}/{{PbI}}_{2}\right]-E\left({{PbI}}_{2}\right)-N\times E\left({ion}\right)$$

where $E\left[{\left({ion}\right)}_{N}/{{PbI}}_{2}\right]$ represents the energy of *N* ions located on the surface or the in-plane edges of PbI_2_ nanosheets, while $E\left({{PbI}}_{2}\right)$ and $E\left({ion}\right)$ denote the energy of the PbI_2_ nanosheet and the energy of the ion, respectively.

### Supplementary information

Supplementary Information

Peer Review File

Description of Additional Supplementary Files

Supplementary Movie 1

Supplementary Movie 2

Supplementary Movie 3

## Data Availability

Relevant data supporting the key findings of this study are available within the article and the Supplementary Information file. All raw data generated during the current study are available from the corresponding authors upon request.
