# Supplementary material for: Growth of millimeter-sized 2D metal iodide crystals induced by ion-specific preference at water-air interfaces
Source: Nat Commun. 2024 Apr 12;15:3185. doi: 10.1038/s41467-024-47241-4 (PMC11014996; doi:10.1038/s41467-024-47241-4)
Supplement: Supplementary file 1 — Supplementary Information [file 41467_2024_47241_MOESM1_ESM.pdf]

Supplementary Information for

Growth of millimeter-sized 2D metal iodide crystals induced by ion-specific preference at water-air interfaces

Jingxian Zhong[1, 2, #], Dawei Zhou[1, 2, #], Qi Bai[3, #], Chao Liu[1, 2], Xinlian Fan[1], Hehe Zhang[1], Congzhou Li[1], Ran Jiang[1], Peiyi Zhao[1], Jiaxiao Yuan[1], Xiaojiao Li[3], Guixiang Zhan[1], Hongyu Yang[1], Jing Liu[1], Xuefen Song[1], Junran Zhang[1], Xiao Huang[1], Chao Zhu[2], Chongqin Zhu[3, *], Lin Wang[1, *]

[1]School of Flexible Electronics (Future Technologies) & Institute of Advanced Materials (IAM), Key Laboratory of Flexible Electronics (KLOFE), Jiangsu National Synergetic Innovation Center for Advanced Materials (SICAM), Nanjing Tech University (Nanjing Tech), Nanjing 211816, China.

[2]SEU-FEI Nano-Pico Center, Key Lab of MEMS of Ministry of Education, School of Electronic Science and Engineering, Southeast University, Nanjing 210096, China.

[3]College of Chemistry, Key Laboratory of Theoretical & Computational Photochemistry of Ministry of Education, Beijing Normal University, Beijing 100875, China.

[#]These authors contributed equally to this work

*Email: cqzhu@bnu.edu.cn, iamlwang@njtech.edu.cn

This PDF file includes:

Supplementary Figures
Supplementary References

Supplementary Figures

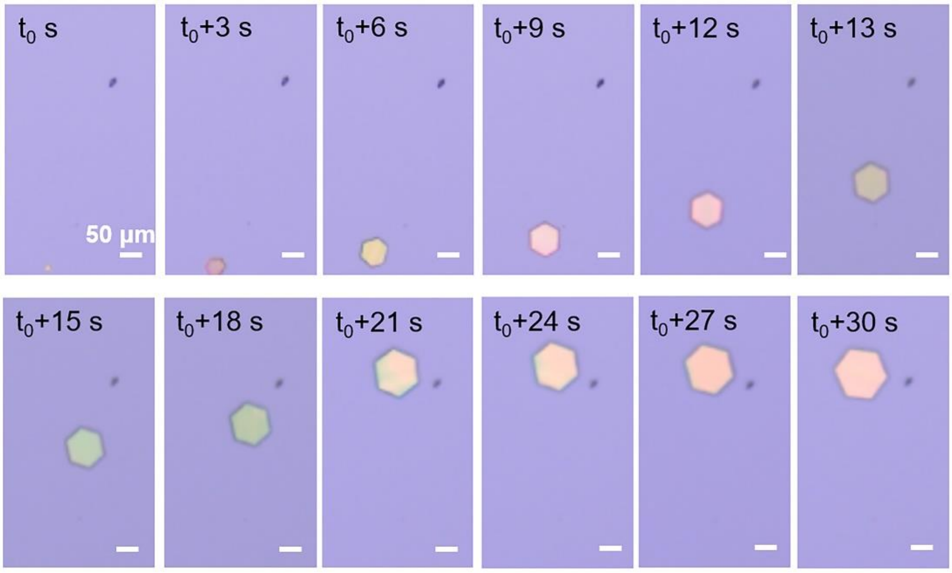

Supplementary Fig. 1 | Snapshots of PbI_2 growth at the water-air interface (t_0≈200 s).

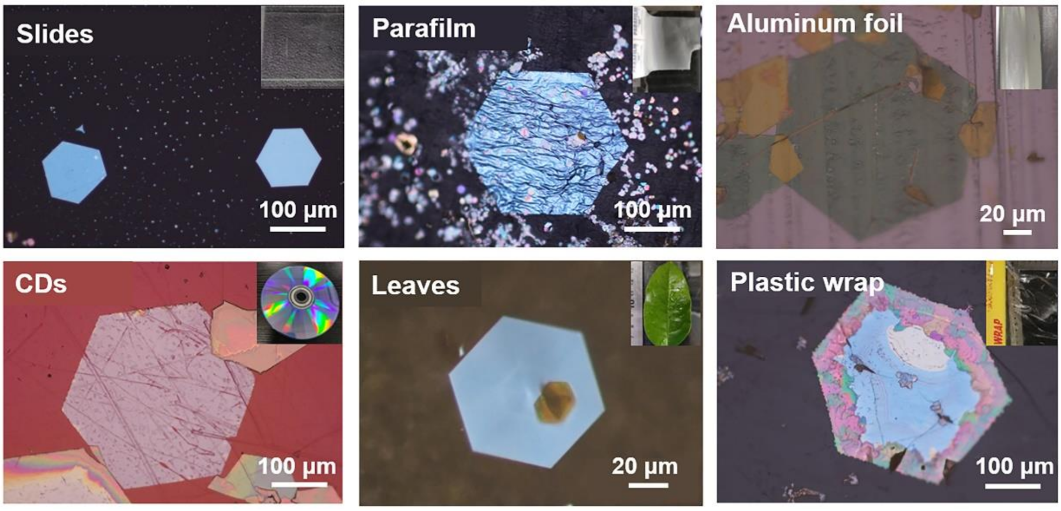

Supplementary Fig. 2 | PbI_2 crystals grown on the slides, parafilm, aluminum foil, CDs, leaves, and plastic wrap.

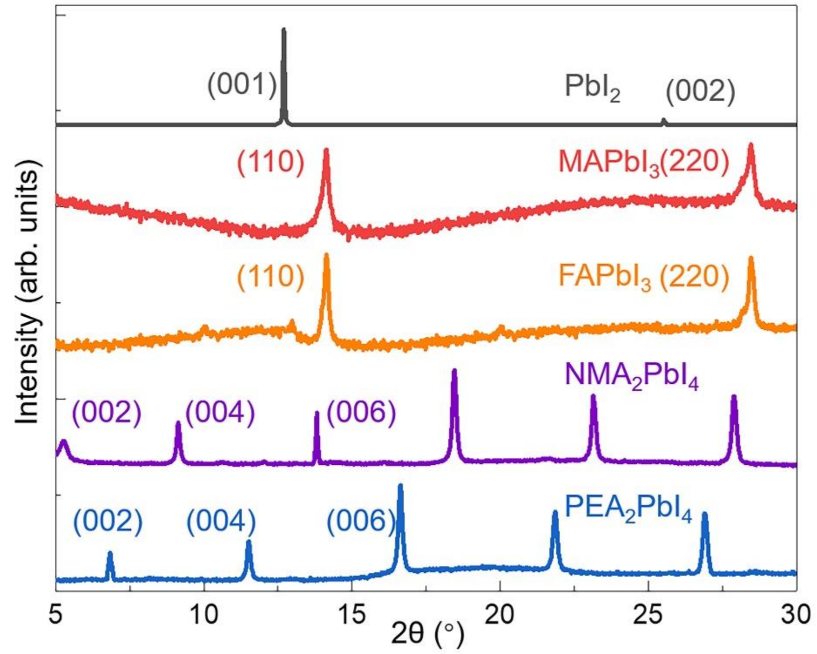

Supplementary Fig. 3 | X-ray diffraction pattern results of PbI_2 and various perovskite nanosheets ($MAPbI_3$, $FAPbI_3$, NMA_2PbI_4, and PEA_2PbI_4) [1-3]**.**

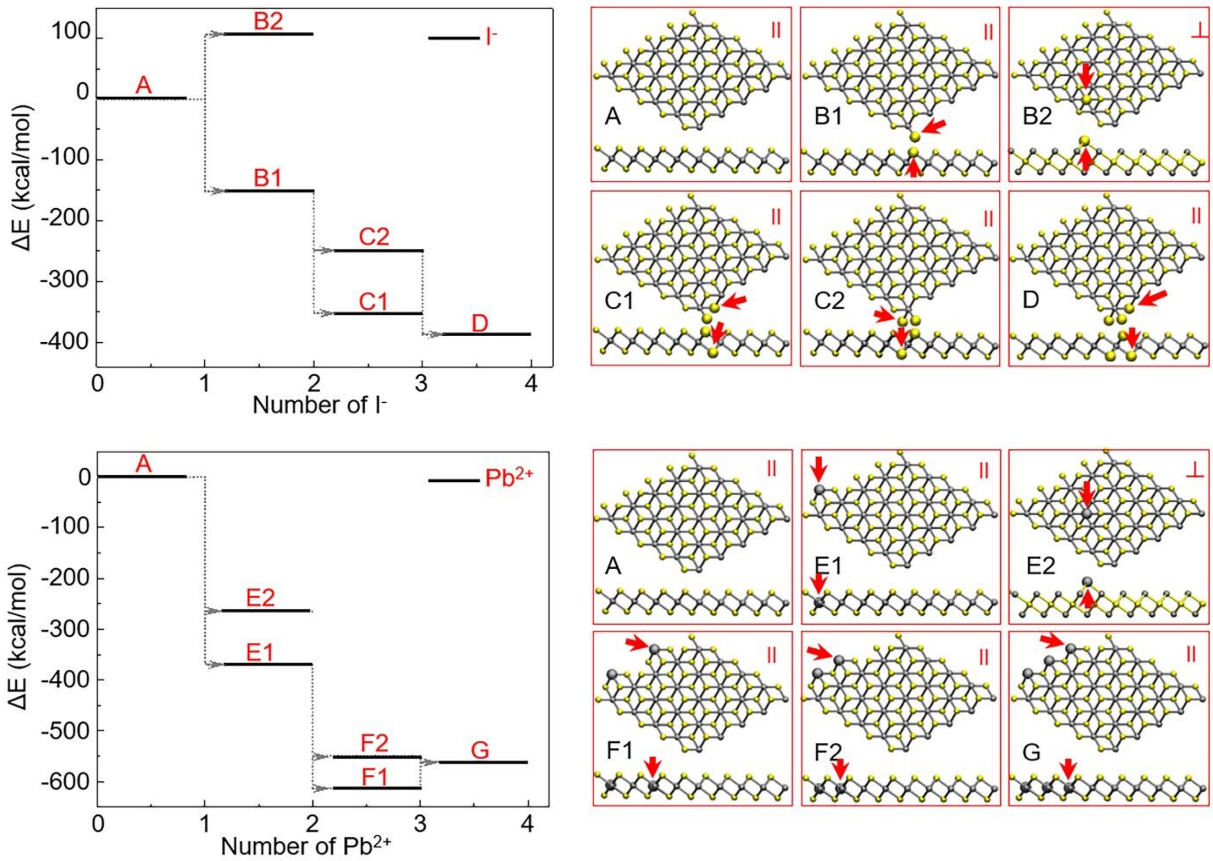

Supplementary Fig. 4 | Energy diagram of the stepwise growth of I^- ions on the nanosheet, where A represents the initial nanosheet. B represents the energy required for adding an I^- ion, with B1 indicating lateral growth and B2 vertical growth. C represents the continuous growth of an I^- ion based on the energy profile B. C1 and C2 correspond to two different lateral growth modes. D depicts the energy diagram for further growth of an I^- ion based on C.

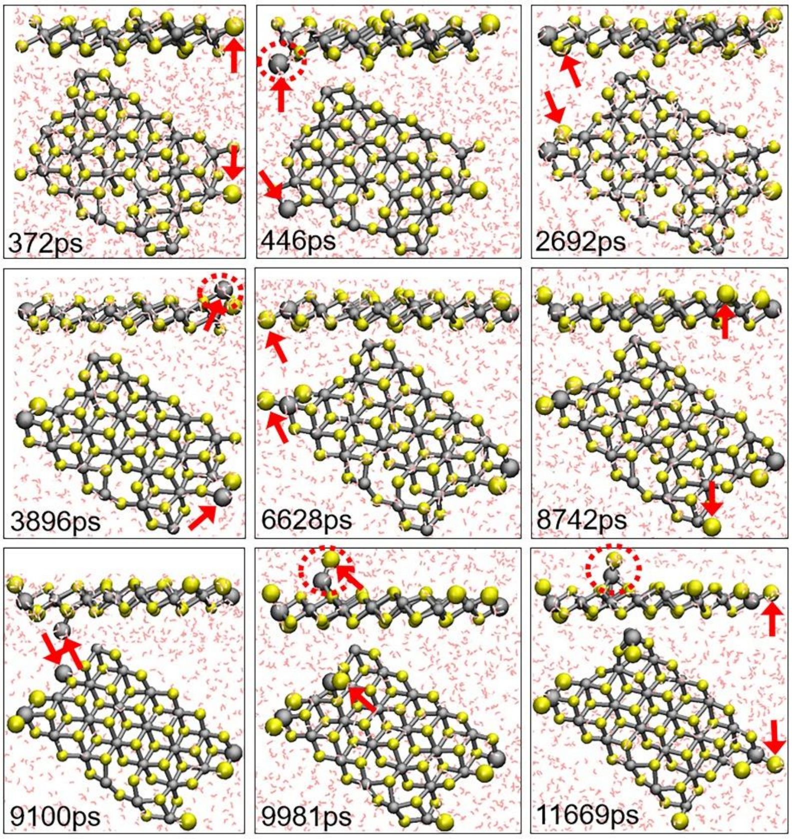

Supplementary Fig.5 | Time-lapse snapshots from molecular dynamics simulations illustrating the growth of the PbI_2 nanosheet in the bulk water, akin to Fig. 2f. The small grey and yellow spheres depict the Pb and I atoms of a pre-existing PbI_2 nanosheet, respectively. The large grey and yellow spheres represent the Pb and I ions initially dissolved in water and are marked with arrows, respectively. Dashed circles denote the vertical growth of ions in the water solution, while the remaining elements depict the horizontal growth of ions in the water solution.

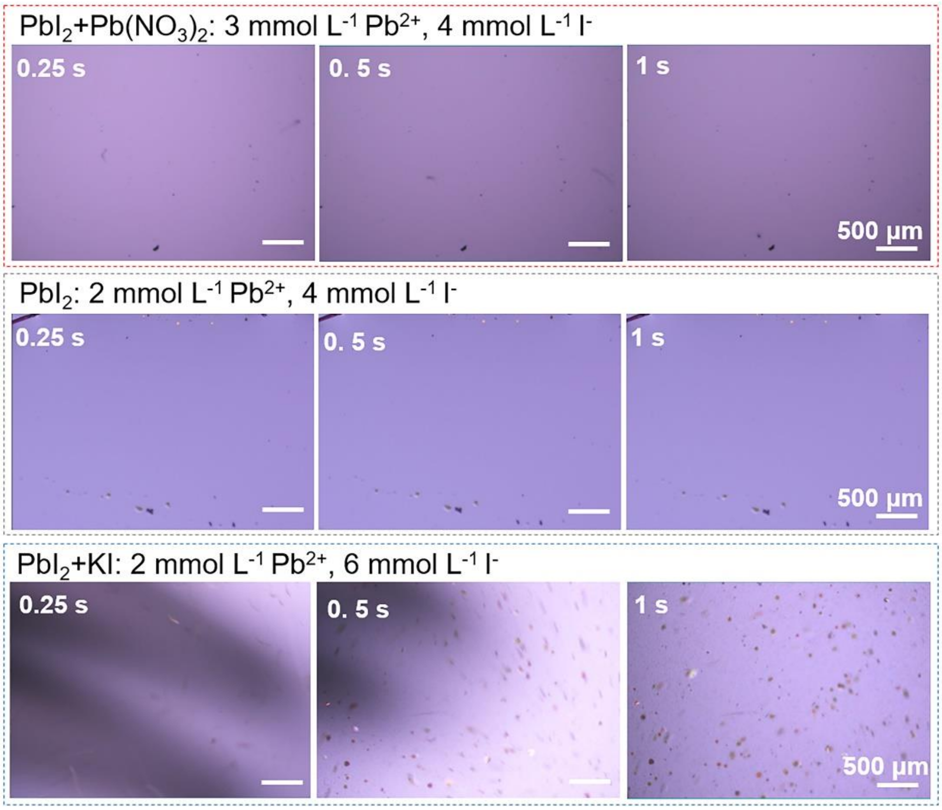

Supplementary Fig. 6 | Snapshots of PbI_2 growth with the addition of $Pb(NO_3)_2$ and KI.

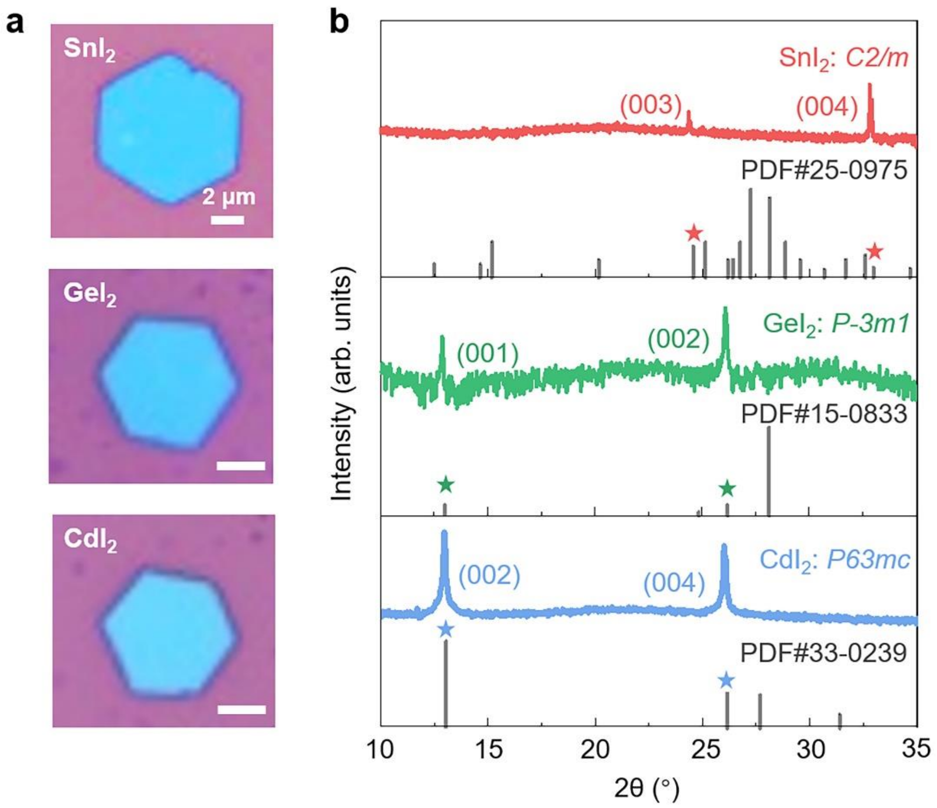

Supplementary Fig. 7 | Water-air interfacial growth of other metal iodides. Optical images (a) and XRD results (b) of SnI_2, GeI_2, and CdI_2**.**

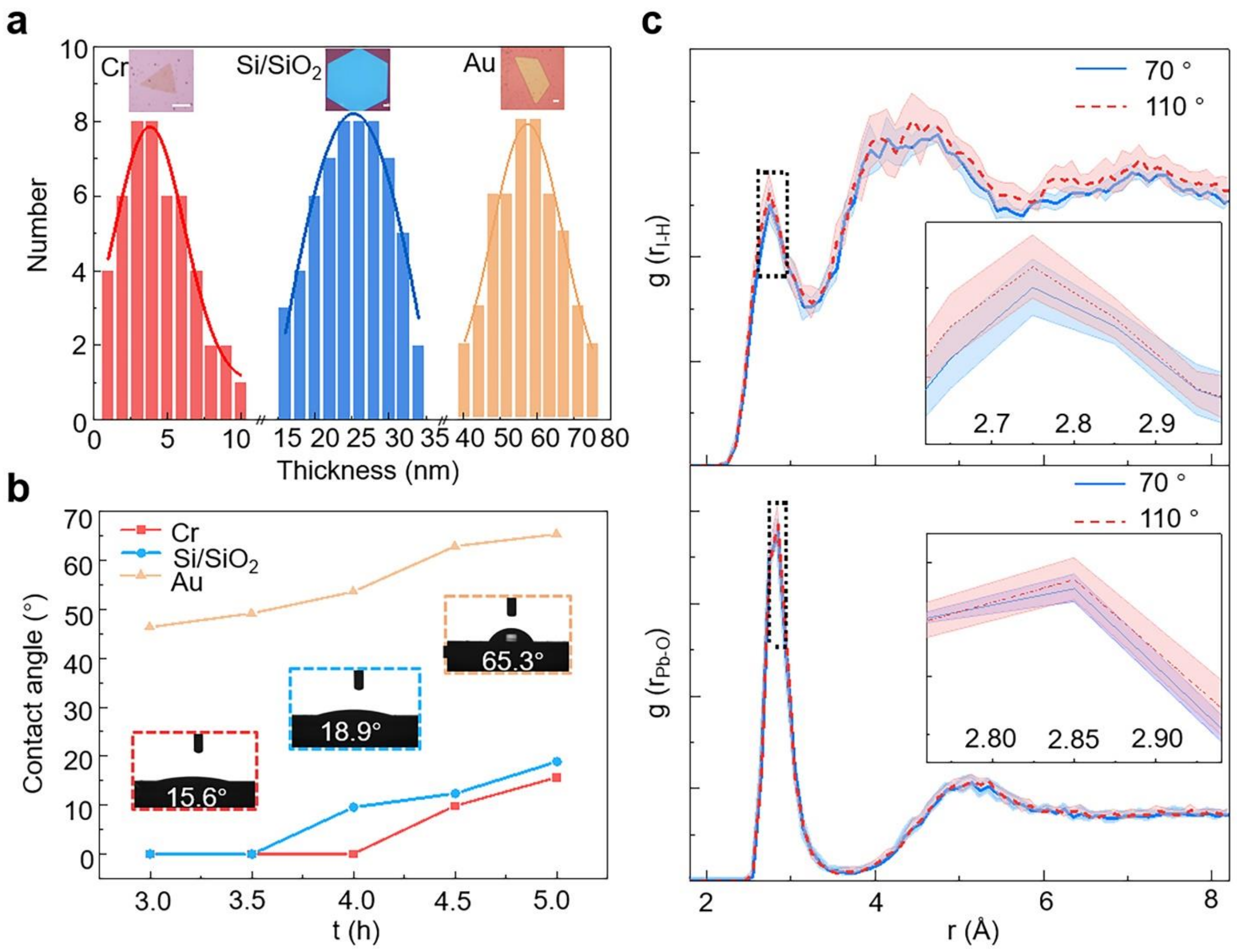

Supplementary Fig. 8 | Effect of solution contact-angle on PbI_2 growth. (a) Statistical distribution of PbI_2 nanosheet thicknesses using different substrates. The solid lines are normal distribution fitting of the experimental data. **(b)** Variation in the contact angle of PbI_2 aqueous solution on different substrates over time. **(c)** Hydration numbers of Pb^{2+} and I^- ions in cases with different contact angles. By analyzing the integral area, a contact angle of 110° corresponds to a larger integral area, indicating a higher number of hydrations of Pb^{2+} and I^-, and thus closer to the bulk phase. In contrast, a contact angle of 70° corresponds to a smaller integral area, indicating fewer hydrations and a closer proximity to the interface.

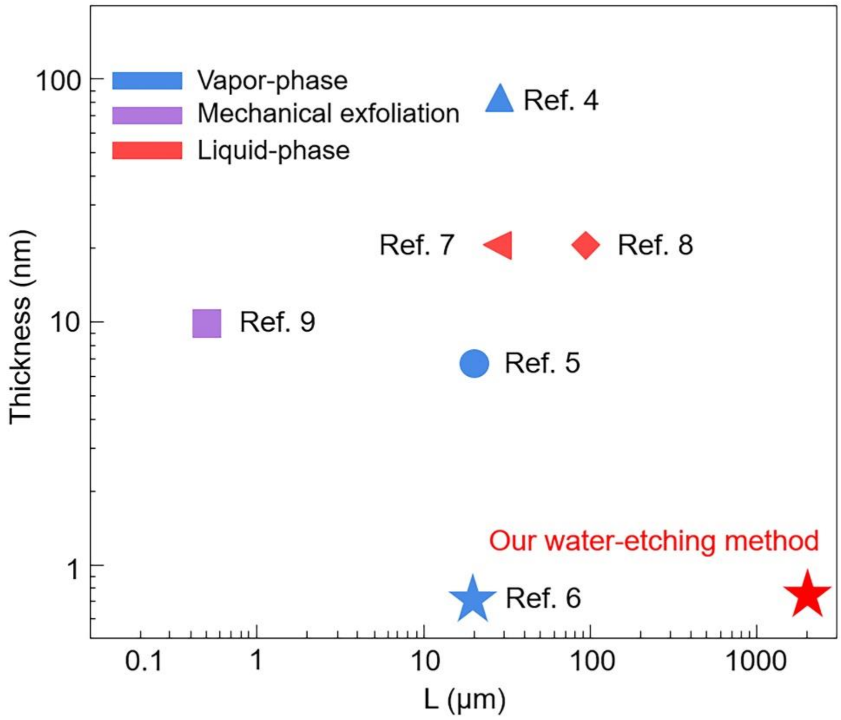

Supplementary Fig. 9 | Comparison of the lateral size (L) and thickness of PbI_2 single crystals prepared using previous methods and our methods [4-9].

Supplementary References

1. Du, K. Z. *et al.* Two-dimensional lead(II) halide-based hybrid perovskites templated by acene alkylamines: crystal structures, optical properties, and piezoelectricity. *Inorg. Chem.* **56**, 9291-9302 (2017).
2. Liu, J. *et al.* Two-dimensional $CH_3NH_3PbI_3$ perovskite: synthesis and optoelectronic application. *ACS Nano* **10**, 3536-3542 (2016).
3. Sun, Y. *et al.* Engineering the phases and heterostructures of ultrathin hybrid perovskite nanosheets. *Adv. Mater.* **32**, 2002392 (2020).
4. Lan, C. *et al.* Large-scale synthesis of freestanding layer-structured PbI_2 and $MAPbI_3$ nanosheets for high-performance photodetection. *Adv. Mater.* **29**, 1702759 (2017).
5. Zhong, M. *et al.* Flexible photodetectors based on phase dependent PbI_2 single crystals. *J. Mater. Chem. C* **4**, 6492-6499 (2016).
6. Zhong, M. *et al.* Large-scale 2D PbI_2 monolayers: experimental realization and their indirect band-gap related properties. *Nanoscale* **9**, 3736-3741 (2017).
7. Xiao, H., Liang, T. & Xu, M. Growth of ultraflat PbI_2 nanoflakes by solvent evaporation suppression for high-performance UV photodetectors. *Small* **15**, 1901767 (2019).
8. Zheng, W. *et al.* High-Crystalline 2D Layered PbI_2 with Ultrasmooth Surface: Liquid-Phase Synthesis and Application of High-Speed Photon Detection. *Adv. Electron. Mater.* **2**, 1600291 (2016).
9. Wangyang, P., Sun, H., Zhu, X., Yang, D. & Gao, X. Mechanical exfoliation and Raman spectra of ultrathin PbI_2 single crystal. *Mater. Lett.* **168**, 68-71 (2016).
