# Supplementary material for: Growth of millimeter-sized 2D metal iodide crystals induced by ion-specific preference at water-air interfaces
Source: Nat Commun. 2024 Apr 12;15:3185. doi: 10.1038/s41467-024-47241-4 (PMC11014996; doi:10.1038/s41467-024-47241-4)
Supplement: Supplementary file 3 — Description of Additional Supplementary Files [file 41467_2024_47241_MOESM3_ESM.pdf]

Description of Additional Supplementary Files

File Name: Supplementary Movie 1
Description: The interfacial preference of PbI_2 nanosheet, the video was generated by classical molecular dynamics simulation. The monolayer PbI_2 nanosheet will spontaneously move toward the interface, where the PbI_2 nanosheet is initially placed in bulk water. The snapshot structures can be obtained from Fig. 2e.

File Name: Supplementary Movie 2
Description: Molecular dynamics simulations of PbI_2 growth at the water-air interface. The video was generated through classical molecular dynamics simulation. The small grey and yellow spheres represent the Pb and I atoms of a pre-existing PbI_2 nanosheet, respectively. The large grey and yellow spheres represent the Pb and I ions initially dissolved in water, respectively. Snapshot structures can be referenced in Fig. 2f.

File Name: Supplementary Movie 3
Description: PbI_2 growth process in its native environment at 298 K.
